# Genetic mapping of novel QTL for seed protein stability in food-grade soybean (*Glycine max*)

**DOI:** 10.1007/s00122-025-05089-2

**Published:** 2025-11-14

**Authors:** Andrew A. Mitchell, Feng Lin, Heng Ye, Tri Vuong, Zixiang Wen, Biructawit Tessema, Randall Laurenz, Raju Thada Magar, Henry T. Nguyen, Dechun Wang

**Affiliations:** 1 Department of Plant and Soil Science, Michigan State University, East Lansing, MI 48912 USA; 2 Division of Plant Science and Technology, University of Missouri, Columbia, MO 65211 USA; 3 Fisher Delta Research Extension and Education Center, University of Missouri, Portageville, MO 63873 USA

## Abstract

**Key message:**

Two novel quantitative trait loci associated with soybean protein content stability were identified on chromosomes 10 and 18. Haplotype analysis showed these to significantly improve stability without protein content penalty.

**Abstract:**

Soybean seed protein content is a complex physiological trait under polygenic control and significant genotype by environment interaction. Protein content is largely influenced by ambient atmospheric temperature at pod-filling, with increased temperatures enhancing seed protein accumulation. The identification of genomic regions associated with protein content stability will facilitate an increased understanding of seed development physiology and assist in the development of more broadly adapted food-grade soybean cultivars. In this work, 210 recombinant inbred lines were derived from the intraspecific cross of the high protein accession BARC_*-*_6 (PI 555396), and the low protein MSU breeding accession E14077 for the investigation of quantitative trait loci associated with protein content and protein content stability across multiple years and test locations. Indices for static protein content stability were used to estimate genome by environment interactions across Northern and Southern soybean production regions. Composite interval mapping returned one stable major effect QTL associated with protein content on chromosome 20 explaining approximately 20.7% of phenotypic variation. Two novel QTLs associated with absolute protein stability were detected on chromosomes 10 and 18, explaining approximately 8.6% and 7.6% of phenotypic variation, respectively. SNP-based haplotype analysis showed simultaneous favorable effects on protein content and stability when desirable alleles for these QTL were pyramided. These results will serve as a valuable tool for the molecular breeding of food-grade soybean cultivars harboring elevated protein content coupled with improved stability across varied environments, thus addressing a key challenge in meeting the global rise in soybean protein demand for both livestock feed and human consumption.

**Supplementary Information:**

The online version contains supplementary material available at 10.1007/s00122-025-05089-2.

## Introduction

Soybean seed (*Glycine max*) is a major global food product, representing the single largest dietary protein source and the most substantial segment of International agricultural trade (Gale et al. 2019). Among agriculturally relevant leguminous crops, soybean is distinguished by its elevated protein content and high-quality essential amino acid profile, making it particularly well suited for the production of livestock feed and human consumables (Cahoon et al. 2010). Relative to all major crop and livestock products, soybean grain is among the most efficient protein and calorie sources per acre when accounting for agricultural inputs. This efficiency positions soybean as a uniquely favorable candidate for future food production in both resource rich and limited systems (Humane Party Report 2019). Generally, soybean cultivars are classified by their end-uses, with ‘feed-grade’ varieties used for animal production and oil extraction, and ‘food-grade’ varieties used for human food products such as tofu, temphe, soy-milk, natto, and miso. Feed-grade seed typically contains 20% oil and 40% protein, while food-grade cultivars contain 45% protein and exhibit additional quality traits such as seed uniformity, clear hilum, yellow seed, reduced anti-nutritional factors, high water absorption potential, and favorable texture, among other end-use specific traits (Jegadeesan and Yu 2020). While the majority of US soybean acreage is planted in feed-grade cultivars, growing awareness of soybean’s nutritional benefits has driven US demand for soybean-derived human food products fivefold in recent years, with this trend expected to continue through the coming decades (Kour et al. 2023; Friedman and Brandon 2001). The breeding of US food-grade soybean cultivars of improved seed quality traits is therefore gaining importance.

Among quality traits associated with food-grade soybean, seed protein content is of particular significance due to its crucial role within the manufacture of most soy-based foods (Jegadeesan and Yu 2020). In soybean, protein content is a quantitatively inherited trait under polygenic control. Within single environment trials, heritability values for seed protein have been shown to routinely exceed 85%, indicating high-effect genetic control under favorable conditions (Tajuddin et al. 2003; Brummer et al. 1997). As the number of test environments increase and become more diverse, heritability is often depressed, with temperature, soil moisture, pest and pathogen pressure, and nutrient availability all affecting final protein development (Wu et al. 2024; Shi et al. 2023; Patil et al. 2017; Arslanoglu et al. 2011; Wolf et al. 1982). These environmental factors, however, do not influence finished protein equivalently across genotypes, with the presence of significant genotype by environment interactions (GEI) well established in soybean populations (Wu et al. 2024; Shi et al. 2023; Abdelghany et al. 2021; Rodrigues et al. 2014; Rotundo and Westgate 2009; Novikova et al. 2018; Nascimento et al. 2010). Recently, performance trials of 91 commercial elite commodity and food-grade cultivars conducted at 9 Michigan locations showed significant GEI for protein content, with both low (< 40%) and high (> 45%) protein content varieties expressing crossover interactions (Laurenz and Wang 2023). Selection of genotypes exhibiting high protein content coupled with minimal GEI metrics across subtle and more extreme environmental fluctuations is therefore an important component of food-grade soybean improvement programs where meeting minimum protein content standards is imperative. Furthermore, as climatological shifts associated with anthropogenic global warming are projected to severely threaten global food security throughout the remainder of the twenty-first century, the initiative of developing soybean germplasm of improved physiological stability will become only more relevant to adequate global agricultural production (Magnan et al. 2019; Malhi et al. 2021).

To-date, genetic analysis efforts within diverse soybean populations have identified major quantitative trait loci (QTL) for protein content across the majority of the 20 linkage groups (Jegadeesan and Yu 2020; Patil et al. 2017). Although the demonstration of QTL stability across multiple environments is required for confirmation and publication, little effort has been made to explore the genetic basis of protein stability itself in soybean, and QTL by environment interactions are often left unexamined. As a result, to our knowledge, there exists no reported significant QTL for protein content stability in soybean. Among publications which have included the explicit quantification of protein stability in soybean, use of these metrics for genetic association analysis have been ignored or unreported. In 2024, perhaps the first exploration of the underlying mechanisms of GEI for soybean seed protein content was conducted by Wu et al. (2024). In this report, 449 genetically diverse soybean accessions were examined for seed protein stability and 9 additional seed quality traits recorded across 7 environments located within the state of Arkansas, US. Protein stability was estimated using Shukla’s stability variance, though was omitted from the QTL mapping efforts. Furthermore, statistical evidence was provided suggesting the independent genetic control of protein content and stability, as derived from a random forest model. This exclusion of stability metrics from genetic association interrogation reflects a general trend broadly observed within similar works. The genetic control of physiological stability has not gone entirely unexplored, however, and evidence exists to suggest at least partial allelic control of dynamic and static stability for traits of high and moderate heritability in many species including *G. max* (Happ et al. 2021; Lozada and Carter 2020; Xavier et al. 2018; Bouchet et al. 2016; Lacaze et al. 2009; Wu 1998; Lin and Binns 1991; Bains 1976). In soybean, Happ et al. (2021) detected a total of 106 QTL for grain yield when comparing several mixed model and traditional dynamic stability metrics in a GWAS conducted using 213 lines grown across 11 environments. Each approach returned distinct significant peaks, supporting the applicability of various metrics for yield stability interrogation and improvement. These findings strongly suggest the potential existence of additional, exploitable genetic mechanisms for the control of moderate to high heritability traits such as seed protein.

The key objectives of this study were therefore to (i) examine the response spectrum of static seed protein stability metrics within a population of RILs segregating for protein content grown across diverse environments, (ii) test the applicability of these metrics for QTL identification, determining the relative genetic location of significant loci associated with static stability and fixed protein content, and (iii) identify and select individuals of exceptional static and dynamic protein stability and protein content for additional efforts toward the development of broadly adapted food-grade cultivars. Together, these objectives aim to help address modern agricultural challenges associated with changing climates and increasing global food demands through the coupling of favorable GEI response patterns and elevated protein contents within commercial soybean.

## Materials and methods

### Population development

A population of RILs (F7 generation) segregating for protein content was developed using the single seed descent method from an intraspecific cross made in 2017 between the lines BARC-6 (51% protein) and E14077 (39% protein) at the Michigan State University Agronomy farm in Ingham County, MI. BARC-6 is a conventional, maturity group III cultivar derived from the high protein by high protein cross CX797-21(4) x D76-8070(3) made at the Beltsville Agricultural Research Center in Beltsville, Maryland (Leffel 1992). E14077 is a low protein, conventional, maturity group 2.4 cultivar derived from U03-300134 × E07051 made at Michigan State University. In addition to high oil content, E14077 shows stable SCN resistance and returned above-average yield scores in 2022 MSU variety performance trials, making it useful for genetic mapping and traditional breeding initiatives. For this study, a total of 573 initial F3:4 lines were developed and 210 used for final mapping, denoted as the population 170,137.

The BARC-6 × E14077 RIL population was grown during the 2019, 2020, 2021, and 2023 seasons in Ingham County, MI (42.7099 N, 84.4713 W). During the 2022 and 2023 growing seasons, the population was grown at the University of Missouri Agronomy research station in Boone County, MO (38.9092 N, 92.2742 W). In 2023, each trial consisted of a subset of the original population representing the 60 most extreme protein phenotypes (30 high/30 low) planted for protein QTL validation efforts. During the 2019 season, leaf tissue was randomly collected from 218 F2:4 individuals and both parental lines for DNA isolation and 6 K SNP chip data collection. Field trials conducted in 2020, 2021, 2022, and 2023 were carried out under randomized complete block design (RCBD) using two replications, while 2019 trials were grown under a partially replicated augmented design. As the lack of replication among RILs in the 2019 trial precluded stability metric estimation and reliable BLUP calculation, data derived from the 2019 trial were omitted from QTL and Haplotype analysis.

### Experimental sites

South-Central Michigan and North-Central Missouri represent diverse growth environments, as well as highly productive commercial soybean regions. Boone County, Missouri is characterized by humid, subtropical growing seasons with typical summertime temperatures ranging between 24 and 35 degrees Celsius, and an average annual precipitation of 112 cm. Ingham County, Michigan is characterized by temperate, warm growing seasons with summertime temperatures ranging between 18 and 30 degrees Celsius, and average annual precipitation of 110 cm. Generally, commercial soybean cultivars sown in North/Central Missouri fall within maturity groups III-IV, while Mid-Michigan varieties span groups I-II. Despite apparent segregation for maturity present within the 170,137 population described here, all individuals selected for mapping were amenable to timely harvest in both Michigan and Missouri trials.

### Genotyping and QTL analysis

Fresh trifoliate leaf tissue was collected from 218 RIL individuals and both parental lines in Ingham County, MI. Samples were freeze-dried at -80 C prior to DNA extraction using the hexadecyltrimethylammonium bromide (CTAB) method as described previously (Kisha et al. 1997). Both parental lines and all individuals were subjected to SNP genotyping by SoySNP6k array using an Illumina Infinium HD BARCSoySNP6K Beadchip and the Illumina iScan system at the Soybean Genetic Lab of Michigan State University, East Lansing, MI (Illumina, San Diego, CA) (Song et al. 2020). Results were recorded and analyzed using the Illumina GenomeStudio software. Following standard quality control methods, a total of 1,566 polymorphic markers were identified and used for linkage map construction using Joinmap (v4.1) (Ooijen 2006), yielding 20 linkage groups representing the 20 G.max chromosomes. SNP filtering was performed at a call-rate threshold of 0.75, resulting in 210 total samples suitable for mapping. All QTL analysis was conducted using the composite interval mapping (CIM) method within WinQTL Cartographer 2.5 (Wang et al. 2012) under forward and backward stepwise regression and a walk speed of 1.0 cM. Significance thresholds were established using 1000 permutations at *a* = 0*.*05 according to standard statistical practices established by Churchill and Doerge (1994) for the reduction of Type I error. This choice of permutation number balances computational requirements with statistical precision and represents the *de-facto* standard for linkage mapping. QTL positions were determined by their logarithm of the odds (LOD) peak locations, and approximate 95% confidence intervals for QTL positions were estimated using standard linear interpolation under a stepwise LOD range of 1.00 according to conventional statistical practice (Li 2011). The percentage of variance explained by each QTL in proportion to total phenotypic variation (*R*^2^) and additive effects were each estimated within WinQTL Cartographer 2.5. All QTL reported here were found to exceed genome-wide LOD threshold values, unless explicitly stated otherwise. For ease of reporting, QTL identified at markers Gm20.19781743, Gm18.57517100, and Gm10.49447586 have been given the abbreviated distinctions *qtlP.20*, *qtlS.18*, and *qtlS.10*, respectively.

Allele frequencies for each significant QTL (*qtlP.20, qtlS.18, qtlS.10*) were calculated for the RIL population 170,137. QTL haplotype effects on protein content and protein content stability were quantified using fixed effect models and analysis of variance (ANOVA) (*a* = 0.05). Allele and grouped haplotype frequencies were similarly quantified for 6 additional *Glycine* populations using publicly available SoySNP50K data (Song et al. 2013; Brown et al. 2021). Linkage disequilibrium (*D’*, *R*^*2*^ and *P* value) between significant QTL was estimated in TASSEL (Bradbury et al. 2007).

### Seed protein phenotyping

Protein content from all years and locations was measured using the DS2500 near infrared spectroscopy (NIRS) instrument (Foss) on a 13% moisture basis in the Soybean Genetics Lab at Michigan State University using previously established calibration equations (R^2^ = 0.97, SEC/SECV = 0.30/0.33) (Supplemental Table 11). Two replicates of each RIL entry derived from each year were scanned. Approximately, 25 g of sampled finished seed were analyzed for each replicate using a standard 150 mm diameter cup. For all descriptive statistics and QTL analysis of seed protein content independent of stability metrics, best linear unbiased predictors (BLUPs) were used.

BLUPs, broad-sense heritability (*H*^2^), entry-mean heritability (*h*^2^*m*), and predicted phenotypic values were estimated for protein content within and across environments within the R package *metan* using the restricted maximum-likelihood (REML) method (Olivoto et al. 2020). For ANOVA interrogation, genotype and GEI were treated as random effects, with environment and rep by environment interaction treated as fixed effects. Likewise, *metan* was used for statistical analysis of environmental correlation and additive main effect multiplicative interaction (AMMI) modeling. ANOVA for haplotype groups was performed on a one-way basis using the R package lm. For individual marker haplotype analysis, means were compared using Fisher’s protected least significant difference (LSD). All post-hoc tests were conducted at a significance level of 0.05. Residual normality was inspected prior to all statistical analysis and data transformation was not found to be necessary.

### Stability phenotyping

For genetic interrogation of static protein stability, absolute stability was calculated for individual genotypes using the following equations according to Reckling et al. (2021).

1 $$\sigma i = \frac{{s{\text{P}}_{j} (y_{ij} - \overline{y}i)^{2} }}{Nenv}$$

2 $$\sigma_{b} = \sigma_{i} /eBLUP_{g}$$

3 $$\sigma_{\mu } = \sigma_{i} /\mu_{g}$$

where *yij* is the observed protein value of the *i*th genotype in the *j*th environment, *yi* is the mean of the *i*th genotype, and *Nenv* is the number of year-location environments. This index estimates stability independent of population means within and across environments and represents unbiased genotypic variance across environments. According to absolute stability standards, genotypes harboring values equal to zero are said to be perfectly stable, with increases corresponding to stability decay.

For QTL analysis, absolute stability was independently calculated using raw protein data on a rep-basis (*σi*), as well as from eBLUP values (*σb*). Furthermore, absolute stability derived from raw data was adjusted for mean genotypic protein content across all environments by dividing absolute stability scores for each genotype by corresponding genotypic means (*σµ*) as described by Reckling et al. (2021). This was done to account for possible unequal variance due solely to protein potentials, effectively penalizing low protein lines. In conjunction with protein content per se, these three related datasets (denoted as *σi*, *σb*, and *σu*), were used for QTL analysis to test the effects of adjustment, if any, on QTL positions and significance.

In addition to absolute stability, dynamic BLUP-based stability metrics were calculated for the identification of desirable lines showing above-average protein content across year/location environments for continued breeding purposes according to Resende et al. (2007). These metrics included the harmonic mean of genotypic values *(HMGV*), relative performance of genotypic values (*RPGV*), and the harmonic mean of RPGV (*HMRPGV*) (Tables 1 and 2). These metrics were not used for QTL mapping as they fail to capture within-line, unbiased GEI, which was the target of this investigation.

**Table 1:** Protein content (%) descriptive statistics for 218 RILs grown in 2 environments for QTL mapping. 2023 statistics were derived from the 30 highest and lowest protein genotypes as determined by 2020–2022 data

Trait	Environment	Mean	SE	Sd	CV (%)	Minimum	Maximum	Range	H^2^	H_e_^2^
Protein	20MI	44.02	0.12	1.81	4.11	37.87	48.29	10.42	0.83	0.91
Protein	21MI	44.31	0.10	1.49	3.36	39.97	48.77	9.4	0.61	0.76
Protein	22MO	45.83	0.16	2.41	5.26	39.1	51.02	11.92	0.62	0.77
Protein	23MI	45.93	0.36	2.77	6.03	38.61	52.59	13.98	0.87	0.93
Protein	23MO	44.53	0.33	2.55	5.73	40.04	49.22	9.18	0.71	0.83
Protein	23_Pooled	45.28	0.26	2.84	6.29	37.45	52.95	15.5	0.64	0.83
Protein	Pooled	44.92	0.09	2.34	5.21	36.97	52.59	15.62	0.62	0.90
*σ*_*i*_	Pooled	1.079	0.045	0.620	–	0.081	3.110	3.029	–	–
*σ*_*b*_	Pooled	0.986	0.031	0.453	–	0.111	2.247	2.136	–	–
*σ*_*u*_	Pooled	0.024	0.001	0.014	–	0.002	0.069	0.067	–	–

σi, absolute stability as calculated from raw protein data; σB, absolute stability as calculated from BLUP-adjusted protein data; σμ, absolute stability as calculated from genotypic means-adjusted protein data; Sd, standard deviation; CV, coefficient of variation; H2, broad-sense heritability; He2, entry-mean heritability

**Table 2:** Favorable genotypes derived from static and dynamic BLUP model analysis

Entry	Protein (%)	Protein rank	Absolute stability	Absolute stability rank	HMGV	RPGV	HMRPGV	Genotype (*qtlS.10_qtlS.18_qtlP.20*)
077	47.02	17	0.193	5	47.20	1.051	1.069	(–_–_Fav)
176	46.41	28	0.218	7	46.20	1.034	1.046	(Fav_UnFav_Fav)
145	45.48	69	0.112	1	45.45	1.017	1.029	(Fav_Fav_Fav)
035	47.16	15	0.816	83	46.83	1.048	1.060	(Fav_Fav_Fav)
194	45.84	53	0.499	28	45.64	1.021	1.033	(Fav_Fav_Fav)
058	48.95	1	2.247	216	48.16	1.079	1.090	(Fav_Het_Fav)
148	48.77	2	1.781	206	48.16	1.079	1.090	(Fav_Fav_Fav)
70	47.82	3	1.005	120	47.92	1.067	1.086	(Fav_Het_Fav)
241	47.63	8	1.065	130	47.46	1.062	1.074	(Het_–_–)
026	47.61	9	1.741	204	47.34	1.060	1.072	(–_Het_Fav)

Protein (%); BLUP-derived predicted phenotypic values from pooled environment data

HMGV, harmonic mean of genotypic values; RPGV, relative performance of genotypic values; HMRPGV, harmonic mean of RPGV

## Results

### Phenotypic analysis for seed protein content

Protein content variation between and within all year-location environments (20,192,023) showed approximately normal distributions expected for quantitative traits. In 2019, seed protein content ranged from 36.59% to 51.75% with a mean and standard error (SE) of (45.39 ± 0.17%). For 2020, the range was 37.87–48.29%, with a mean and SE of (44.02 ± 0.12%). In 2021, the recorded range, mean, and SE were 39.37–48.77% (44.31 ± 0.11%). Finally, protein content from 2022 showed a range, mean, and SE of 39.1–51.02% (45.83 ± 0.17%). The validation subset consisting of the highest and lowest 30 protein phenotypes grown in 2023 ranged in protein content from 38.61% to 52.6% and showed a mean and SE of (45.92 ± 0.35%) in South-Central Michigan. In North-Central Missouri, the population showed a range of 40.04% to 49.22% protein and a mean and SE of (44.67 ± 0.31%) (Table 1).

### Environmental variance

ANOVA results across locations showed highly significant effects of genotype, environment, and dynamic GEI on protein content. The effect of GEI was found to represent the second largest source of variation behind genotype, explaining approximately 14% of phenotypic variation (Supplementary Table 7). Across all environments, no transgressive segregation was detected for protein content. Broad-sense heritability for predicted protein content across all year/location environments was found to be 0.62, with entry-mean heritability returning a value of 0.90. Individual environment broad-sense heritability values ranged from 0.61 to 0.87, while entry-mean heritabilities from 0.76 to 0.93 (Table 1).

**Fig. 1:**
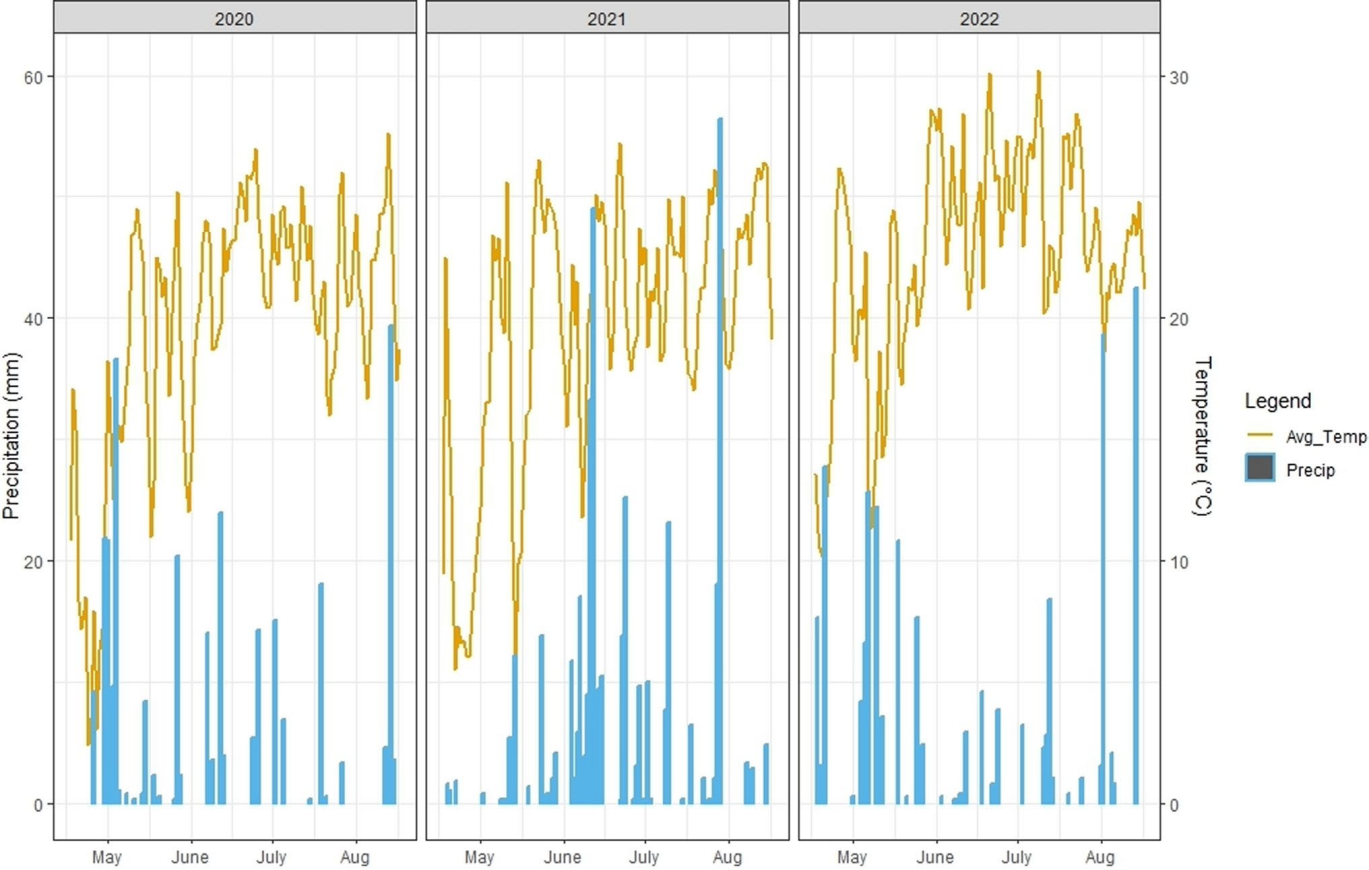
Average daily temperature and rainfall records between May 1st and September 1st across three year-location environments

Multivariate statistical analysis using additive main and multiplicative interaction (AMMI) data and derived biplots also showed genotype, environment, and GEI to all have significant effects on protein content. In addition, significant environmental variation between years and locations was demonstrated by AMMI vector orientation and length (Supplementary Table 9, Supplementary Fig. 1). Furthermore, Pearson correlation tests showed moderate positive correlations between predicted protein content values across year/location environments ranging from 0.66 to 0.78 at a high degree of significance (Supplementary Fig. 3). Weather data further demonstrated adequate environmental variance, with the Missouri site showing significantly elevated average daily temperature (24.43 C) and cumulative precipitation (166.87 mm) during the pod-filling growth period relative to either trial conducted in Michigan (University MS 2025; Missouri 2025) (Fig. 1, Supplementary Table 2). As increased temperatures and available soil moisture have been shown to positively influence seed protein content accumulation in soybean, these factors are likely responsible for a significant portion of the GEI effects observed between sites. Furthermore, the statistically significant elevation of mean protein content observed in 2022 where all 218 RIL individuals were tested at the Missouri site is likely due in part to these favorable conditions (Table 1, Supplementary Table 8).

### Stability analysis

**Fig. 2:**
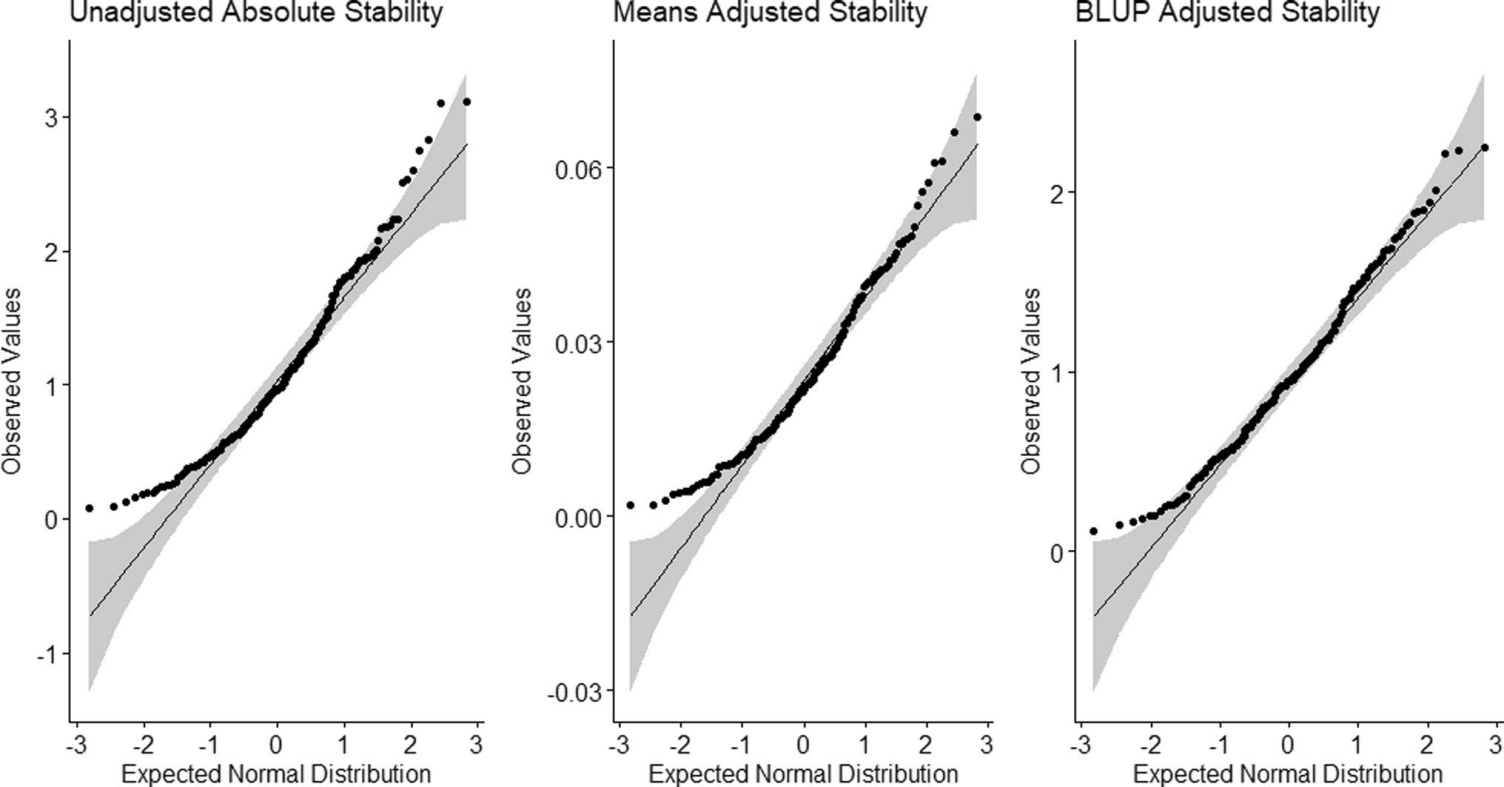
Quantile–quantile probability plots for three stability metrics calculated from 218 RILs grown over three year-location environments. Results indicated relative normality suitable for QTL analysis

Static stability as calculated from raw data, means-adjusted raw data, and BLUP-adjusted data, each showed approximately normal distributions expected for polygenic traits (Fig. 2). Absolute stability derived from raw data (*σi*) ranged from 0.081% to 3.11% and showed a mean and SE of (1.08% ± 0.039%). BLUP-adjusted absolute stability (*σb*) ranged from 0.11% to 2.25% and showed a mean and SE of (0.98% + /0.031%). Means-adjusted absolute stability (*σu*) ranged from 0.77 to 7.01 and showed a mean and SE of (3.11 ± 0.078) (Table 1). As the stability metrics *σi* and *σb* are reported in percentage protein, each can be interpreted as the absolute GEI effect on protein content across genotypes, with smaller stability scores indicating less GEI effect. A stability score of zero therefore indicates no absolute GEI effect, while higher scores indicate significant genotypic sensitivity across environments. For reference, the largest absolute stability score measured in this work was 3.11% in RIL-105, which showed protein content values ranging from 42.5 to 49.6%. The lowest absolute stability recorded was 0.081% in RIL-006, which showed protein contents ranging from 43.62 to 43.86%. In addition, the relationship between absolute protein content stability and raw mean protein was investigated using Pearson correlation analysis and returned a correlation value of 0.37 at *p* < 0.0001, indicating a weak positive relationship. As expected, relative protein stability was found to dampen this correlation slightly, returning a score of 0.35 following mean protein content adjustment. Coefficients of variation were omitted for stability metrics as their proximity to zero yielded unrepresentative results, suggesting exaggerated variance.

### Desirable breeding RILs

Among the 218 RILs phenotyped within this work, top performing lines based on tandem selection of static protein stability and protein content profiles were identified. Likewise, high performing lines based on dynamic BLUP-derived stability metrics were also identified. As described previously, HMGV, RPGV, and HMRPGV were individually inspected and used to rank genotypes relative to overall performance and dynamic stability across environments. Notably, of the seven lines selected which held complete SNP data at each QTL identified within this work, all showed favorable allele status at *qtlP.20*, while nine showed favorable alleles at both *qtlS.10* and *qtlS.18*. The remaining line showed favorable allele only at *qtlS.10* and *qtlP.20* (Table 2). Based on this phenotypic and genotypic analysis, several individuals have been ultimately selected for use as parental lines within the Michigan State Soybean breeding pipeline. These lines serve as valuable genetic resources for specialty high protein cultivar development as well as further investigation into the genetic basis of protein content performance and plasticity.

### QTL analysis

CIM analysis of QTLs associated with BLUP predicted protein content across pooled year/location environments returned single significant loci located on chromosome 20 at marker Gm20.19781743 (89.77 cM). This QTL showed a LOD score of 13.3 and a percent of total phenotypic variance explained (PVE) of 19.3% (Table 3). Analysis of individual year/location environment trials returned an identical chromosome 20 QTL to that seen within pooled year data. This locus was detected in every environment tested and returned LOD scores ranging from 10.34 to 14.79, and PVE values from 17.49 to 23.71%. This QTL was therefore considered stable and was included in subsequent SNP-based haplotype analysis. In addition to the major QTL on chromosome 20, several minor QTLs for protein content were identified within data derived from 2020 Michigan field trails. These included loci on chromosomes 01 and 14 showing LOD and PVE values of 3.87/5.06% and 3.79/5.08%, respectively. Although significant, neither was found to be stable across years or within pooled environment data and were therefore omitted from additional analysis (Table 3).

**Table 3:** Summary of CIM QTL analysis results for 210 RILs evaluated in 3 environments and 60 extreme protein phenotype RILs tested at two environments

Environment	Trait	Chromosome	QTL name	Position (*cM*)	95% Confidence interval	Peak marker	LOD threshold	LOD	Additive effect	R^2^ (%)
MSU-20	Protein	**20**	***qtlP-20***	**89.77**	**89.71–89.81**	**Gm20_19781743**	**3.65**	**12.94**	**0.77**	**0.20**
MSU-20		01	*qtlP-01*	40.74	**38.27–41.23**	Gm01_5453015	3.65	3.87	0.38	0.05
MSU-20		14	*qtl1P-14*	36.89	**34.33–38.22**	Gm14_4889916	3.65	3.79	0.37	0.05
MSU-21		**20**	***qtlP-20***	**89.77**	**89.58–89.87**	**Gm20_19781743**	**3.69**	**10.34**	**0.45**	**0.17**
MSU-23		**20**	***qtlP-20***	**89.43**	**89.35–89.85**	**Gm20_8185857**	**3.51**	**3.99**	**1.19**	**0.19**
MSU-23		14	***qtl2P-14***	3.5	**3.44–3.63**	Gm14_587754	3.51	3.98	1.12	0.15
MO-22		**20**	***qtlP-20***	**89.77**	**89.56–89.83**	**Gm20_19781743**	**3.58**	**14.79**	**0.96**	**0.24**
MO-23		**20**	***qtlP-20***	**89.34**	**89.28–89.52**	**Gm20_18531300**	**3.78**	**4.86**	**1.05**	**0.16**
Pooled (23)		**20**	***qtlP-20***	**89.39**	**89.26–89.52**	**Gm20_7927513**	**3.7**	**8.23**	**1.44**	**0.27**
Pooled (20,21,22)		**20**	***qtlP-20***	**89.77**	**89.70–89.85**	**Gm20_19781743**	**3.69**	**13.29**	**0.72**	**0.19**
Pooled (20,21,22)	*σ*_*i*_	10	*qtlS-10*	89.38	**87.63–91.68**	Gm10_49447586	4.03	4.34	0.20	0.09
Pooled (20,21,22)	*σ*_*B*_	18	*qtlS-18*	93.63	**93.40–94.69**	Gm18_57517100	3.65	3.71	0.14	0.07
Pooled (20,21,22)	*σ*_*μ*_	10	*qtlS-10*	89.38	**87.90–91.30**	Gm10_49447586	4.00	4.81	0.00	0.10

σi, absolute stability as calculated from raw protein data; σB, absolute stability as calculated from BLUP-adjusted protein data; σμ, absolute stability as calculated from genotypic means-adjusted protein data; LOD, logarithm of the odds; R2, proportion of variance explained by genetic effects. Bold characters show QTL which were stable across all environments tested. Italic values indicated the names assigned to QTLs within this report

For protein QTL validation, the 60 most extreme protein phenotypes (30 highest/30 lowest) were grown in 2023 at both locations simultaneously. For pooled 2023 data, CIM analysis again detected a major QTL at marker Gm20.19781743 for seed protein, showing a LOD of 8.23 and a PVE of 26.94%. Individual MSU and MO environment data also returned this loci, where it was found to show LOD and PVE scores of 4.00/18.58% and 4.86/16.12%, respectively. In addition, the Michigan 2023 trial returned a unique significant large effect QTL on chromosome 14 at marker Gm14.587754 (3.5 cM), showing a LOD of 3.98 and PVE of 15.09%. This loci was not detected in any previous trials despite its high degree of significance and major effect size, and was unique to the chromosome 14 loci detected in 2020. QTL analysis of stability metrics between environments in 2023 was not investigated as selected lines showing extreme protein phenotypes did not accurately represent RILs of extreme stability phenotype. This rendered the limited population unsuitable for reliable QTL interrogation against stability metrics due to the minuscule sample size coupled with an approximately normal distribution of stability trait values.

**Fig. 3:**
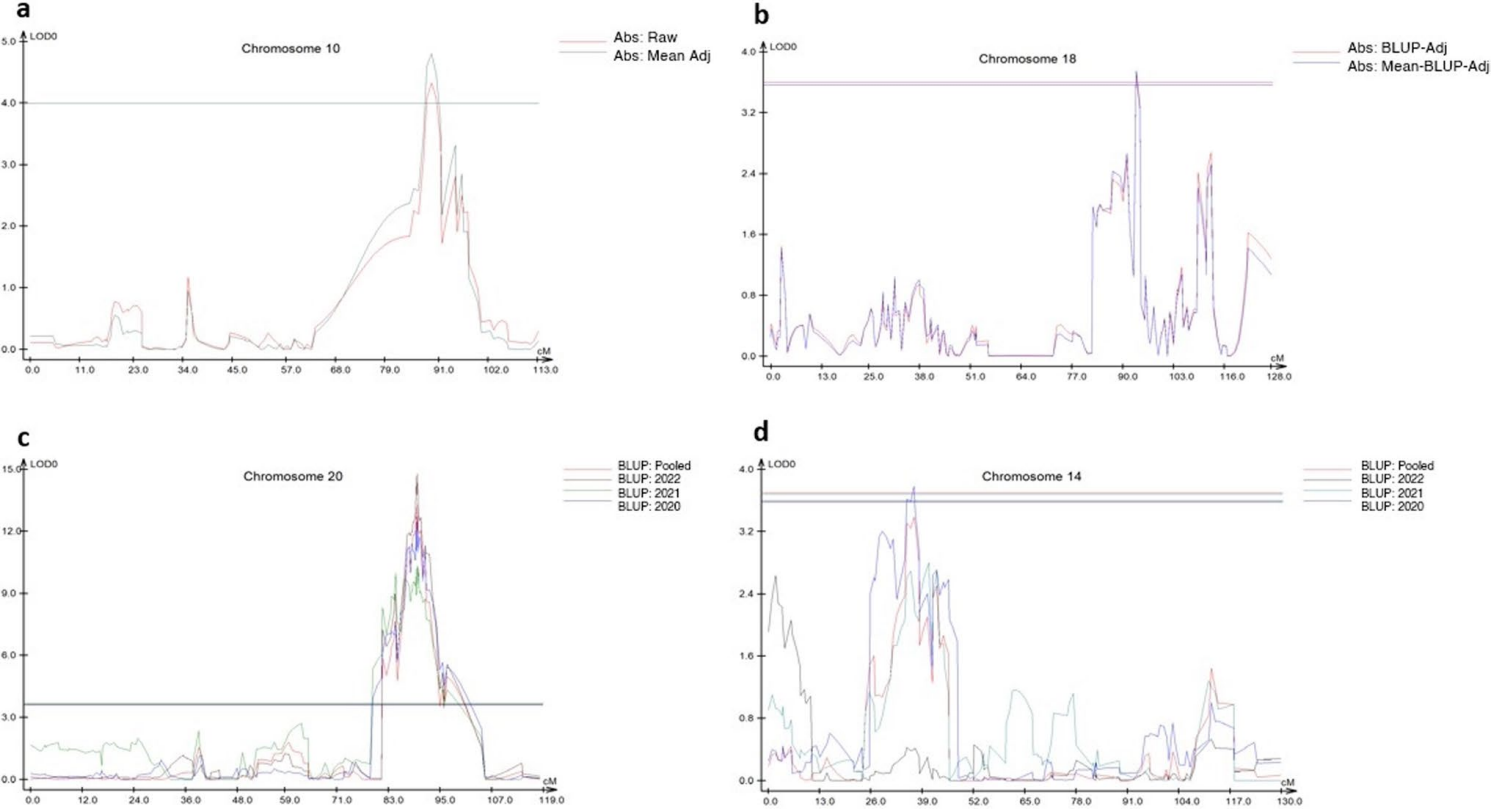
QTL cartographer results depicting significant peaks for stability and protein content metrics. **a** QTL Peaks obtained using CIM on for unadjusted absolute stability across all year/location environments; **b** QTL Peaks obtained using CIM on for BLUP- and Means-adjusted absolute stability across all year/location environments; **c** QTL peaks obtained using CIM on chromosome 20 for BLUP predicted protein content across individual year/location environments and pooled environment data; **d** QTL peaks obtained using CIM on chromosome 14 for BLUP predicted protein content across all year/location environments. Significant peak obtained from 2020 Michigan trial

CIM analysis for absolute protein stability was conducted individually using unadjusted, BLUP-adjusted, and means-adjusted protein data. Overall, two significant QTL were identified, with loci coincidence observed between two stability metrics. Absolute stability analysis using unadjusted raw data (*σi*) returned a single, unique QTL on chromosome 10 at marker Gm10.49447586 (89.40 cM) which explained 9.32% of phenotypic variance and showed a LOD score of 4.34. For BLUP-adjusted absolute protein stability across all year/location environments (*σb*), a single QTL was identified on chromosome 18 at marker Gm18.57517100 (93.33 cM). This locus showed a LOD of 3.71 and PVE of 6.75% (Table 3, Fig. 3). Means-adjusted absolute stability *σu* returned a single significant QTL identical to that identified by *σi* at marker Gm10.49447586, though at a marginally heightened LOD value of 4.81 and PVE of 10.39% (Table 3, Fig. 3). Notably, the chromosome 18 QTL identified by *σb* was also associated with *σi*, though at a depressed LOD value of 3.31, which was found to be significant only at the chromosomal basis despite being the second highest LOD value returned for the trait.

### SNP-based haplotype analysis

To determine the individual and pyramiding effect of the QTL reported here, joint SNP-based haplotype analysis was conducted using the peak markers Gm20.19781743, Gm18.57517100, and Gm10.49447586. Using SNP reads, 210 genotypes were grouped into corresponding allele classes for variance analysis. ANOVA was conducted to analyze haplotype group means by BLUP-adjusted protein content and BLUP-adjusted absolute stability as these were considered the most practical and accurate phenotypic datasets for phenotypic selection. All ANOVA tests were performed using fixed effect models and post-hoc tests conducted using a confidence interval of 0.95 and an alpha value of 0.05.

Individual analysis of *qtlP.20* favorable alleles showed a highly significant haplotype effect for protein content (*p* = 6*.*13*e−*15), and a reduced though significant effect for absolute stability (*p* = 0*.*017) when genotypes homozygous for either SNP allele were examined. As expected, BARC-6 was shown to be the donor parent for the high protein allele, the presence of which resulted in moderately dampened stability. Homozygous genotypes for the BARC-6 derived allele at *qtlP.20* (*n* = 80) showed a mean protein content of 45.64% ± 0.15%, and mean absolute stability of 1.09% + /0.05%. Genotypes homozygous for the E14077 allele at this loci (*n* = 75) showed a mean protein content of 43.86% ± 0.15% and mean absolute stability of 0.86% + /0.05% (Supplementary Table 4).

For *qtlS.18*, marginally significant allele effects were shown for absolute stability (*p* = 0*.*015), though this QTL, when examined alone, was not shown to significantly effect protein content (*p* = 0*.*65). E14077 was found to provide the favorable allele at these loci. Genotypes homozygous for the low protein cultivar E14077 derived SNP (*n* = 54) had a mean absolute stability of 0.94% ± 0.05%, and a mean protein content of 44.85% ± 0.19%. Genotypes homozygous for the BARC-6 *qtl.18* allele (*n* = 69) had a mean absolute stability of 1.14% ± 0.07%, and a mean protein content of 44.73% ± 0.19% (Supplementary Table 5).

ANOVA results for *qtlS.10* were not dissimilar to those of *qtlS.18*, showing a significant effect on absolute stability (*p* = 0*.*004), though no effect on protein content (*p* = 0*.*76). Again, E14077 provided the favorable stability allele at these loci. Genotypes homozygous for the E14077 allele at *qtlS.10* (*n* = 79), showed a mean absolute stability of 0.95% ± 0.06%, and a mean protein content of 44.61% ± 0.21%. Homozygous genotypes harboring both BARC-6 alleles at these loci (*n* = 76) had a mean absolute stability of 1.21 ± 0.07% and a mean protein content of 44.70% + /0.17% (Supplementary Table 6).

**Fig. 4:**
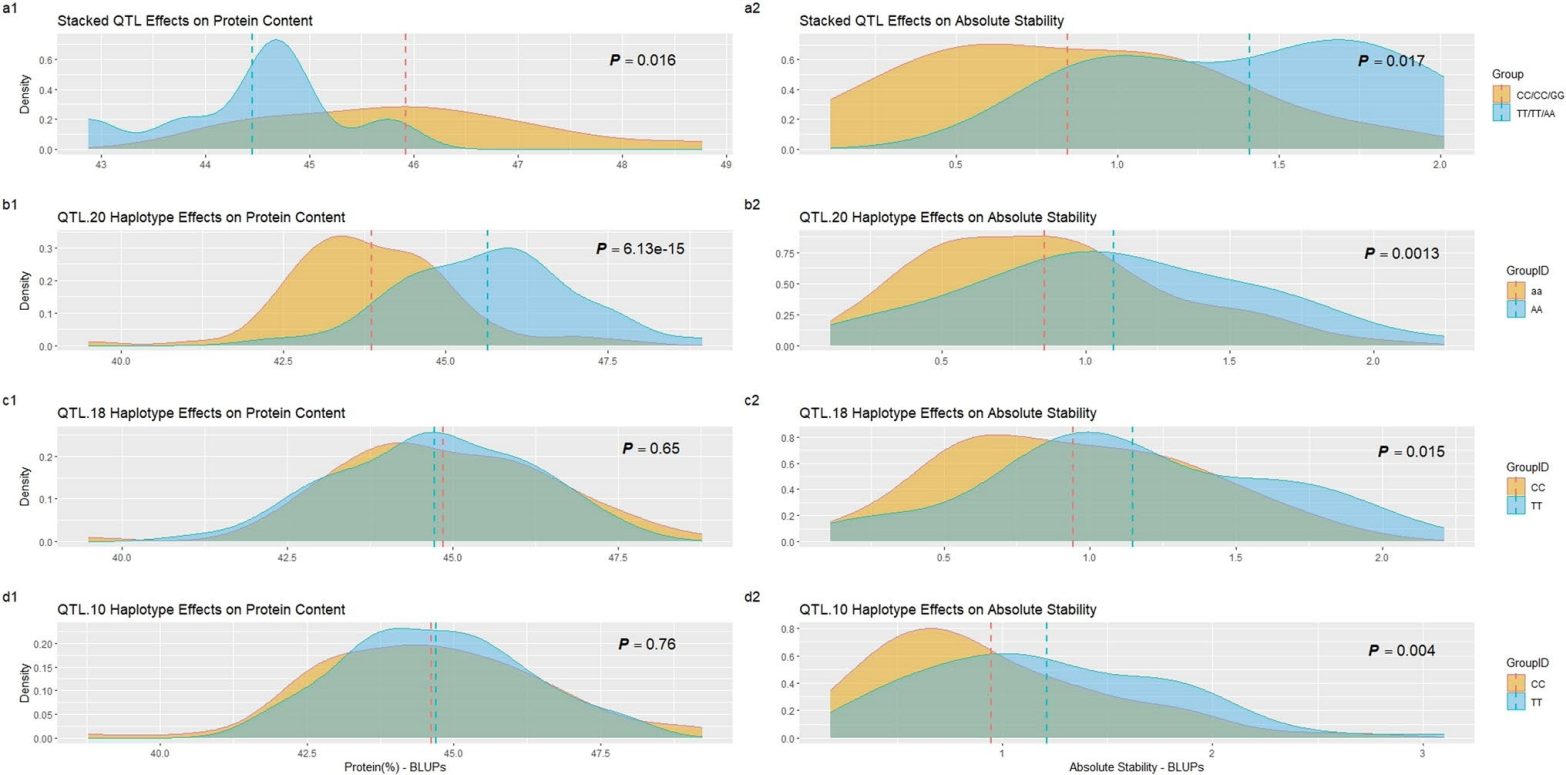
Protein content and BLU adjusted absolute stability of favorable and unfavorable haplotype groups for individual and stacked QTL from combined year/location environment data. **a1** and **a2** effect of the 3 stacked QTL on protein content absolute stability; **b1** and **b2** effect of the Gm 20 protein QTL on protein content and absolute stability; **c1** and **c2** effect of Gm 18 stability QTL on protein and stability; **d1** and **d2** effect of Gm10 stability QTL on protein and stability. Individual and pyramided haplotype effects indicate utility for stability improvement without protein content penalty

To test the potential application of the QTL identified within this work for practical soybean improvement, a joint haplotype analysis was performed using combined interrogation of pyramided favorable *(CC/CC/GG)* and unfavorable *(TT/TT/AA)* alleles inferred from individual haplotype ANOVA. As anticipated, stacked haplotype groups showed significant effects on BLUP predicted protein content (*p* = 0*.*016), as well as BLUP-adjusted absolute stability (0.017). Genotypes harboring favorable alleles at all three loci (*n* = 13) showed a significantly elevated mean protein content of 45.92% ± 0.36% relative to genotypes containing unfavorable alleles (*n* = 7), which showed a mean protein content of 44.45% ± 0.34%. These represented the highest and lowest mean protein contents among all four allele groups, respectively. This trend was present for BLUP-adjusted absolute stability as well, with favorable genotypes showing an improved mean absolute stability of 0.84% ± 0.13% relative to unfavorable genotypes, which showed a mean absolute stability of 1.41% ± 0.17% (Fig. 4). Again, these results represent the statistically highest and lowest mean stability scores among all allele groups tested (Fig. 4).

### Haplotype diversity

To further investigate the relative frequency of favorable SNP-haplotypes identified in this work, additional genotypes were analyzed as described above. Included in this effort were 93 elite MSU cultivars, 20,098 publicly available *G.max* accessions, 1200 publicly available high protein (> 45%) *G.max* accessions, 1164 publicly available low protein (< 40%) *G.max* accessions, 96 reference *Glycine soja* accessions, 96 *G.max* landrace lines, and the 210 RILs generated from this work. All publicly available SNP data gathered was originally generated using the SoySNP50K SNP array, while elite MSU-line marker data was derived using the SoySNP6K SNP array (Song et al. 2020, 2013; Brown et al. 2021).

Within the 20,098 *G.max* accessions tested, linkage disequilibrium analysis was conducted between *qtlP.20, qtlS.18,* and *qtlS.10,* with weak to moderate linkage shown between *qtlP.20* and *qtlS.10* (*D’* = 0.428, *r*^*2*^ = 0.035). No significant linkage disequilibrium was detected between any other QTL pairs (*D’* < 0.15). Haplotype analysis showed stacked favorable alleles at each of *qtlP.20, qtlS.18,* and *qtlS.10* to occur at a frequency of 0.187 (*n* = 3,751).

The 1,200 high protein accessions analyzed at these loci showed a significantly decreased frequency of lines harboring the stacked favorable QTL haplotype, down to 0.068 (*n* = 82). Inversely, the 1164 low protein lines showed a significantly higher frequency of 0.298 (*n* = 347). The 96 landrace accessions showed a frequency of 0.073 (*n* = 7), while the 93 MSU elite germplasm showed a frequency of 0.022 (*n* = 3) for favorable stacked alleles (Supplementary Table 3. The 210 RILs used for QTL mapping returned favorable haplotypes at a frequency of 0.081 (*n* = 17), and among the 96 *G.soja* accessions, favorable haplotypes were not recovered in any individual accession. This was driven by the low frequency of the *qtlP.20 and qtlS.10* favorable alleles in *G.soja*, which were shown to be 0.125 (*n* = 12), and 0.052 (*n* = 5), respectively. Favorable allele frequency at *qtlS.18* in these accessions was found to be 0.36 (*n* = 35).

The low occurrence of the favorable *qtlP.20* haplotype within *G.soja* stood in dramatic contrast to all other lines tested. Among the 96 landrace accessions, the favorable *qtlP.20* haplotype existed at a frequency of 0.88 (*n* = 85), and was shown to be 0.97 (*n* = 90) among the 93 elite MSU cultivars (Table 4). Within the high protein accessions, the *qtlP.20* favorable haplotype occurred at a frequency of 0.768 (*n* = 922), while in low protein lines this frequency unexpectedly increased to 0.903 (*n* = 1051). Among the RIL group, a decreased frequency of 0.381 (*n* = 80) was found. The favorable *qtlS.10* haplotype likewise saw a significant shift between *G.soja* and landrace lines, with an increase in frequency from 0.049 (*n* = 5), to 0.54 (*n* = 52). This frequency decreased in MSU elite lines to 0.14 (*n* = 13), though was elevated in all other populations (Table 4). Favorable haplotype frequencies at *qtlS.18* saw less significant change, occurring at a rate of 0.36 (*n* = 35) in *G.soja*, 0.30 (*n* = 29) among landraces, and 0.25 (*n* = 23) among MSU elite lines. The overall mean and standard deviation of frequencies for favorable individual haplotypes at each of *qtlP.20, qtlS.18,* and* qtlS.10* across all groups were 0.797 ± 0.195, 0.334 ± 0.099, and 0.381 ± 0.091, respectively. The mean and standard deviation of frequencies for the stacked favorable haplotype was found to be 0.127 ± 0.091 (Table 4).

**Table 4:** Favorable allele haplotype frequencies across 7 populations representing diverse backgrounds and protein content classes. High protein PIs filtered from all PI lines based on average seed protein above 45%. Low protein PI lines filtered from all PI lines based on average seed protein below 40%. MSU Elite lines include cultivars showing various seed composition phenotypes

Population	Population size	Favorable haplotype frequency			
		*qtlP.20/qtlS.18/qtlS.10*	*qtlP.20*	*qtlS.18*	*qtlS.10*
PI lines	20,080	0.187	0.874	0.344	0.553
High protein PI lines	1200	0.07	0.768	0.202	0.452
Low protein PI lines	1164	0.298	0.903	0.540	0.552
MSU elite lines	93	0.021	0.971	0.256	0.141
*G*. *max* landraces	96	0.104	0.885	0.302	0.542
*G*. *soja* accessions	96	0.00	0.125	0.365	0.052
(BARC-6 × E14077) RILs	210	0.081	0.381	0.329	0.376
Mean	–	0.127	0.797	0.334	0.381
SD	–	0.091	0.195	0.099	0.191

## Discussion

Soybean represents the single most abundant protein source globally and the second most widely grown crop in the USA. Despite its relatively high heritability, significant GEI exposes even elite food-grade cultivars to potential major fluctuations in finished seed protein content within and across environments (Wu et al. 2024; Nascimento et al. 2010; Laurenz and Wang 2023). With heightened climactic uncertainly becoming an ever-present reality, GEI for all crops is expected to increase over the remainder of the current century. Soybean protein content is particularly susceptible to climatic shifts, as it has been shown to be largely influenced by moisture levels and ambient temperatures at key development times (Wu et al. 2024; Laurenz and Wang 2023). Deviation in either of these environmental factors outside those considered optimal for proper soybean seed development has significant impacts on final protein content in most cultivars studied. As extreme weather events including heatwaves, prolonged drought, and flooding due to excessive rainfall are forecast to increase in frequency, the development of physiologically stable soybean cultivars will become only more crucial to sustained domestic and International agricultural productivity as we progress through the century. To accomplish this goal, molecular tools facilitating the realization of soybean crop profiles showing heightened protein content and minimized environmental sensitivity will need to be developed. The QTL identified within this work represent an initial step in this direction and a promising route toward deeper elucidation of the underlying mechanisms driving GEI of seed quality traits in soybean.

In the present study, the potential genetic control of several stability metrics were investigated by QTL mapping of a RIL population developed from the high protein line BARC-6 and the low protein line E14077. The complete population was evaluated in two locations over three years, representing three total environments. Following initial QTL results, validation trials were conducted on the sixty most extreme protein content phenotypes in both Michigan and Missouri. Following QTL mapping, a haplotype analysis was conducted to test individual and combined effects of the three QTL identified on protein content and absolute stability. From these tests, several desirable genotypes were selected as resources for additional research and breeding efforts.

### Environmental variance

For individual and pooled environment data, relatively normal distributions for protein content and absolute stability values were observed. Across all year/location environments, field trials conducted in Missouri in 2022 showed a significantly increased average protein content relative to all other year/locations (45.83%) (Table 1). Given the elevated mean summertime temperatures and elongated growing seasons experienced in Boone County, MO relative to Ingham County, MI, this trend is consistent with previous works establishing a positive correlation between seed protein and increased day/night temperatures at the pod-filling stage (Wolf et al. 1982; Novikova et al. 2018; Sobko et al. 2020; Wang et al. 2015). Missouri trials in 2022, however, showed a slightly depressed broad-sense heritability of 0*.*62 relative to values observed among additional environments examined in this work (Table 1). This is likely a result of favorable conditions for protein development driving phenotypic variation, as illustrated by a significantly increased mean, SD, and CV at this location. As the variable physiological ceilings and genetic constraints present within RIL populations produce differing responses to optimal environments, pronounced mean–variance scaling under favorable conditions was an expected outcome. Further evidence of this trend is provided by the observed correlation between protein content and stability decay, indicating heteroscedasticity among individuals within and across locations.

Field trials conducted in MI during the 2021 growing season likewise exhibited a relatively dampened *H*^2^ of 0*.*61. Below average end-of-season ambient temperatures and high moisture levels resulted in significant micro-environmental variance. This data, however, was deemed retainable due to reasonable replication, low outlier incidence, and apparent normal distributions for protein content and single trial stability indices.

Further investigation of environmental effects using ANOVA and AMMI methods indicated significance from pairwise and cumulative environment testing (Supplementary Table 8, Supplementary Table 9, Supplementary Fig. 1). In addition, Pearson correlation tests showed moderate correlations between individual datasets which indicated a degree of dissimilarity between year/location environments (Supplementary Fig. 3). As the objective of this study was to test physiological stability across highly varied environments, these results support the presence of adequate variation for the purpose of phenotypic stability analysis despite the use of limited locations.

### Stability index selection

Within the context of plant improvement, phenotypic stability is a complex topic for which there exists a wide array of mathematical and conceptual definitions. The relative similarities and differences between these have been examined and re-examined as the concept has progressed and its measurement evolved in statistical sophistication (Lin et al. 1986; Freeman 1973; Marquez-Sanchez 1973). For stability analysis, the selection of an appropriate definition of stability suitable for the specific objectives outlined is a crucial component to deriving useful and interpretable results. Here, two distinct conceptual categorizations were carefully considered, (i) dynamic, and (ii) static stability (Pour-Aboughadareh et al. 2022).

Generally, *dynamic* stability refers to the quantification of genotypic performance across environments relative to fluctuating population means driven by GEI. These metrics have been reported as useful for interrogating traits of low to moderate heritability such as yield, where consistent performance across diverse conditions is not a reasonable biological expectation (Pour-Aboughadareh et al. 2022; Hill et al. 1998). In contrast, *static* stability refers to the quantification of genotypic performance independent of population means and focuses on identifying individuals demonstrating minimal GEI irrespective of relative population performances (Lin and Binns 1991; Pour-Aboughadareh et al. 2022; Hill et al. 1998; Yue et al. 1997). Static metrics therefore provide a statistical quantification of individual stability without adjustment for rank performance and are therefore practical for the development of cultivars showing broad adaptability. For stability-associated QTL identification, a static approach was considered based on previous works establishing the applicability of such metrics for trait variance analysis and their observable, at least partial genetic control and selectability (Lin and Binns 1991; Hill et al. 1998; Specht et al. 2001).

Previously, several groups have attempted to identify genetic components of stability for yield using a broad range of dynamic indices and models (Happ et al. 2021; Lacaze et al. 2009; Wu 1998; Specht et al. 2001; Sandhu et al. 2021). The strategy of analyzing a wide range of stability metrics was not adopted here, however, as many well-established variance indices were found to be incompatible with the measurement of quality-trait variance across diverse test environments within RIL populations for the explicit purpose of genetic mapping loci associated with GEI. Primarily, this incompatibility stemmed from the reliance on population mean adjustment inherent within most parametric and non-parametric stability quantification methods, rendering them largely ineffective at capturing within-line GEI independent of generalized population trends (Lin and Binns 1991; Lin et al. 1986; Pour-Aboughadareh et al. 2022). Furthermore, many non-parametric analyses tend to forgo the quantification of stability and instead rely on ranking and classification metrics which group genotypes based on like and unlike environmental responses (Lin et al. 1986; Pour-Aboughadareh et al. 2022). These methods, though not directly applicable to the objectives outlined in the present work, are not without utility, and have therefore informed our use of several modified-static approaches in conjunction with a strictly static methodology. For this, a single static index (absolute stability) was ultimately selected and tested against raw, BLUP-adjusted, and within-genotype-means-adjusted protein data. This approach allowed for the examination of stability with and without the consideration of local within-environment variance, as well as generalized macro-environment trends characterized by genotypic means.

Absolute stability, represented by the population standard deviation of protein content observed within a single genotype across test environments, serves as a simple yet informative metric for the quantification of static phenotypic stability for traits of relatively high heritability (Reckling et al. 2021; Pour-Aboughadareh et al. 2022; Hill et al. 1998). This metric, though useful, is not without limitation, as its accuracy is largely dependent on outlier severity and observation number. Furthermore, like many parametric stability indices which attempt to represent multivariate interactions by univariate quantification, absolute stability may be significantly confounded by unequal micro-environment effects when calculated on a rep-basis. In addition, several authors have brought attention to historic breeding trends which have disproportionately favored dynamic stability traits over static in most major crops. As a result, elite cultivars in North American crop species routinely show limited adaptability to narrowed production regions (Lin and Binns 1991; Lin et al. 1986; Pour-Aboughadareh et al. 2022; Hill et al. 1998). This factor becomes important within the present work as increases in mean protein content were significantly (though weakly) associated with lower static stability metrics across environments. The magnitude of this relationship, though not entirely dependent on historic germplasm selection patterns, has likely been influenced by these trends. The observed correlation between improved protein content and decreased stability is not however, a ubiquitous pattern, as several populations of elite soybean cultivars grown across multiple test environments have shown both low and high protein lines to exhibit varying degrees of adaptability and stability metrics (Wu et al. 2024; Nascimento et al. 2010; Laurenz and Wang 2023).

To account for the limitations described here, absolute stability was calculated for raw, BLUP-adjusted, and mean-adjusted protein data. The use of these three datasets allowed for the simultaneous examination of static protein stability at varying degrees of statistical correction. In this way, the effects of noise arising from within-environment variance, as well as unequal genotypic protein response potentials were accommodated at least partially. Surprisingly, QTL mapping results derived from each of these data revealed similar trends in LOD distribution and proportionality. This suggests absolute stability to likely be an appropriate and reliable means of quantifying static stability within the multi-location replicated trials presented here.

Additionally, although the relationship between protein content and stability is diverse and complex, the interplay between these two traits and yield was not investigated within the scope of this work. Previous work has shown highly elevated seed protein (50–57%) to be associated with significant yield reduction in soybean, making this topic of primary interest for future work involving the 170,137-RIL population. It is noteworthy, however, that cultivars of moderate protein improvement (~ 45%) have been developed showing only marginal yield penalty, demonstrating practicality in the decoupling of protein content and yield drag (Burton et al. 1999; Chen et al. 2011). Regardless, the interplay between seed protein, stability, and plant yield will be a crucial topic of investigation for future work both toward genetic characterization and cultivar development.

### QTL mapping

Phenotypic stability is a complex and often unpredictable agronomic quality even for moderately to highly heritable traits. Although challenging, the development of cultivars possessing favorable static stability and performance has been demonstrated previously and is therefore not considered a genetic impossibility even for complex traits such as yield (Reckling et al. 2021; Lin et al. 1986). The discovery and deployment of genetic markers associated with even marginal gains in absolute GEI avoidance would assist the development of cultivars suited to a broadened range of environmental conditions within and across geographic regions.

Here, significant QTLs were identified for both protein content and absolute protein stability. Three total QTLs were identified for protein content. One large effect (> 15%) region was found on chromosome 20 (*qtlP.20*) in and across all years tested, while one small effect (< 5%) region on chromosome 1 (*qtlP.*01) was identified in 2020, as well as within pooled year data. The validation population planted in 2023 at both locations revealed an additional large effect QTL for protein on chromosome 14 (*qtlP.*14).

 Interestingly, despite this QTL’s large effect, it was not detected in any other year/location environments. The chromosome 20 protein QTL was therefore the only loci found to be stable across environments within either population. Analysis of absolute stability using BLUP-adjusted protein content returned a single significant, moderate effect (5–10%) QTL located on chromosome 18 (*qtlS.18*). Surprisingly, analysis against raw and means-adjusted protein content each returned a single, identical small effect QTL on chromosome 10 (*qtlS.10*).

QTLs for protein content have been reported on the majority of soybean chromosomes (Patil et al. 2017). However, nearly all published loci remain untested across diverse germplasm and environments and confer only small to moderate phenotypic effects. Among officially confirmed protein QTL is cqSeed Protein-003 located on chromosome 20, as first reported by Diers and colleagues in 1992. The identification of a stable, large effect QTL on chromosome 20 associated with seed protein content within this work is therefore not an unprecedented finding. It is noteworthy, however, that the QTL reported here showed a relatively dissimilar position (12.9 Mb, 79–103 cM) against the W82.a2.v1 reference genome assembly to that of cqSeed Protein-003 (25.3 Mb, 25–30 cM), indicating that these QTL may in fact be under unique genetic control. Further support for this claim is provided by Ravelombola et al. (2022), who recently reported an additional large effect protein QTL at 45.5 Mb (118–166 cM) on chromosome 20 within a closely related BARC-7 derived RIL population. Though these results are similarly non-localized to those reported by Diers, our validation efforts carried out in 2023 as described previously, support our reported position of *qtlP.20* at 15.9 Mb within the BARC-6 RIL population. Despite these distal positions, genetic ontology and syntenic analysis of these divergent chromosome 20 QTL may reveal conserved genetic ontology and syntenic structure (Leffel 1992). Furthermore, work demonstrating the occurrence of QTL divergence in soybean provides additional evidence to suggest potential genetic homology between protein QTL of more distal positions on chromosome 20 and others (Lestari et al. 2013). Nonetheless, the unique position of *qtlP.20* may in fact suggest it to be a novel QTL controlled by differing causative genetic features. The *qtlP.20* associated peak marker Gm20.19781743 has been shown to occur within a zinc finger CCHC domain-containing protein. This family of proteins is expansive, with highly diverse functionality across species and tissues (Hu and Zuo 2021). Recently, (Li et al. 2017) implicated the zinc finger CCHC domain-containing protein GmZF351 to play a key role in oil accumulation in soybean, suggesting the existence of possible additional ZFCCHC proteins in soybean influencing physiological development. Furthermore, (Fliege et al. 2022) recently concluded by fine mapping and RNAi validation that the most likely causative gene associated with cqSeed Protein-003 is the CCT protein-encoding Glyma.20g085100. Of course, barring fine-mapping and in-depth molecular characterization efforts, explanatory genetic associates of the QTL identified in this work will remain speculative in nature. Regardless of these loci’s possible redundancy, the QTL at marker Gm20.19781743 reported here will certainly facilitate protein content improvement by molecular selection within the BARC-6 derived breeding populations at MSU.

Similarly to *qtlP.20* reported here, the small effect QTL located on chromosome 1 associated with seed protein appears to have been previously reported by Brummer et al. (1997). Interestingly, Brummer and colleagues showed this QTL at position 39.8–41.8 cM to be stable across several years, while in the present work it was identified in a single environment only. Somewhat unsurprisingly, the two BARC-7 derived populations examined by Ravelombola et al. (2022) did not show QTL on chromosome 1 for protein content in any environment tested. The general applicability of *qtlP.01* for protein improvement within the Michigan State University breeding program is therefore low barring it’s identification within potential future trials. This apparent instability and low PVE also precluded its inclusion in further haplotype effect analysis.

In addition to protein content loci, one small effect QTL was identified for absolute stability on chromosome 18 (*qtlS.18*) at 103.3 cM when BLUP-derived predicted protein values were used. This QTL was also detected when raw and means-adjusted protein content was used, though at LOD levels meeting only chromosomal significance thresholds. While no previous reports have documented QTL for static or dynamic stability of seed protein in soybean, several seed protein QTL of varying effect have been detected on chromosome 18 at similar positions to *qtlS.18* reported here (Tajuddin et al. 2003; Brummer et al. 1997; Diers et al. 1992). Ravelombola et al. 2022 identify a significant protein QTL on chromosome 18 within the previously mentioned BARC-7 RIL population, though this position (0*.*00*−*14*.*9* cM*) was distal enough to consider it non-redundant. Considering *qtlS.10* identified from absolute stability using raw and means-adjusted data, a similar trend is observed, with QTL for protein content reported previously at the position reported here (Specht et al. 2001; Chen et al. 2007). As protein content and protein stability were shown to be significantly correlated within this work, it is not unreasonable to expect some degree of cross-talk between QTL identified for either trait. Notably, however, all QTL identified here were trait specific for all datasets tested, with no protein loci identified for stability or *vice-versa*. Despite this loci exclusivity, haplotype ANOVA did show *qtlP.20* to significantly affect absolute stability, though neither *qtlS.18* nor *qtlS.10* could be shown to affect protein content. These results suggest that despite the two trait’s correlation, the genetic basis of this trend within the test population may have been weak enough to avoid identical loci detection between traits. Furthermore, the previous identification of seed protein QTL at similar positions to the stability QTL shown here is not altogether unsurprising considering the close biological association these traits invariably have. Despite this, the failure to identify these QTL for seed protein within this work suggests their potential utility for protein stability improvement independently from protein content itself within the given population. For broader claims regarding generalized stability improvement within diverse populations and test environments, further testing and demonstration of replicability will be necessary. Furthermore, it is worth noting that the introgression of three unique QTL within single elite food-grade lines represents a significant challenge and risk considering the relatively moderate effect sizes of *qtlS.18* and *qtlS.10* and the potential for unfavorable phenotypic consequences related to excessive linkage drag or unanticipated epistatic, QTL x QTL, and QTL x ENV interactions*.* These limitations, however, do not preclude further investigation of the results reported within this work for future soybean improvement. Efforts to validate these QTL within diverse backgrounds and additional environments are currently underway.

### Haplotype significance

To investigate the potential phenotypic effects of the protein and stability QTL reported here by ANOVA, haplotype analysis was conducted using individual and stacked peak markers for each loci. Inspection of *qtlP.20*, *qtlS.18*, and *qtlS.10* was conducted separately for individual effects and in tandem for stacked effects. Unsurprisingly, *qtlP.20* was shown to independently have a significant effect on both protein content and protein stability. This finding is consistent with the established correlation between high protein content and low protein stability within the BARC-6 RIL population studied here. This correlation presents a specific challenge to breeders focused on the simultaneous improvement of protein content and stability, as selection of this QTL alone may increase protein content, though at the cost of dampened static stability. The potential utility of effective markers associated with stability separate from those associated with protein content per se is therefore clearly illustrated by this finding.

The haplotype analysis of *qtlS.18* independently showed no significant effect on protein content, though did show a significant effect on BLUP-adjusted absolute stability. This trend was mirrored by *qtlS.10* when analyzed independently. This finding serves to validate the QTL results reported within this work and may indicate these markers to be potentially useful within the context of molecular assisted protein content stability improvement uncoupled from protein content reduction.

Furthermore, to examine the phenotypic effects of the three pyramided QTL, a presence absence SNP-based haplotype ANOVA was performed. These results showed the stacking of favorable alleles at all three loci to significantly and simultaneously improve protein content and protein content absolute stability. Although the sample sizes were somewhat diminutive, power testing at observed effect sizes indicated their relative suitability for the described analysis. These results demonstrate a promising proof of concept for the simultaneous improvement of soybean protein content and static stability using a marker assisted breeding strategy.

Finally, to assess the relative occurrence of the SNP-haplotypes identified here within a broader collection of germplasm, several diverse populations were examined. These populations included 20,098 publicly available accessions, 1200 accessions of elevated protein phenotype (> 45%), 1164 low protein lines (< 40%), 96 *Glycine soja* accessions, 96 *G.max* landrace lines, 93 elite MSU lines of varying phenotype, and the 210 RILs generated within this work. Each population was examined for favorable haplotype frequencies at *qtlP.20*, *qtlS.18*, and *qtlS.10* both individually and collectively.

From this analysis, favorable haplotypes appeared to exist concurrently at these loci in a relatively small proportion of soybean genotypes, with high protein and elite status conferring no apparent advantage. The largest frequency of occurrence was found, surprisingly, within the low protein accessions (0.298). High protein lines, inversely, showed a frequency of only 0.068, with 82 lines harboring the favorable allele collection. This trend, though unexpected, appeared to be driven less by group differences in *qtlP.20* frequency than by differences in *qtlS.18*. Within both high and low protein groups, *qtlP.20* favorable haplotype frequencies were relatively high, at 0.768, and 0.903, respectively. The high occurrence of the favorable qtlP.20 allele across elite cultivars was expected, as this QTL region has been previously shown to be associated with seed size and yield, and therefore an important haplotype for soybean domestication (Goettel et al. 2022). By contrast, favorable alleles at *qtlS.18* were reduced by more than half in the high protein group relative to the low protein group, dropping from 0.540 to 0.202. This frequency at *qtlS.18* in the high protein group was the lowest observed in any tested. The favorable haplotype frequencies at *qtlS.10* and *qtlS.18* within the low protein group, however, were both among the highest observed (Table 4). Furthermore, the surprisingly high occurrence of the favorable *qtlP.20* haplotype within low protein lines was made less anomalous by the consideration of frequencies observed across all other reference *G.max* groups, which ranged from 0.768, to 0.971 (excluding RILs). It thus appears that within cultivated populations, the favorable haplotype at *qtlP.20* occurs at high frequencies regardless of seed protein content, while *qtlS.10* and *qtlS.18* show significantly higher variation across groups. The high frequency of favorability at *qtlS.10* and *qtlS.18* within the low protein group is also consistent with the RIL population described in this work, as both favorable haplotypes at these loci were shown to be derived from the low protein parent E14077. This parent did not, however, harbor the favorable haplotype at *qtlP.20* which occurred at a high frequency within the low protein reference group.

In addition to the results seen between high and low protein groups, examination of the *G.soja* reference accessions revealed several trends of interest. Within these *G.soja* lines, favorable haplotypes at *qtlP.20* and *qtlS.10* were found to occur at very low frequencies of 0.125 and 0.052, respectively. In contrast, the landrace reference group showed frequencies of 0.885 and 0.542 for these haplotypes. Similar frequencies were identified within the high and low protein groups, as well as the general PI group. Interestingly, however, frequencies at *qtlS.18* showed significantly less variance between *G.soja* and all other groups tested (Table 4). These findings may suggest the *qtlP.20* and *qtlS.10* loci to have played potentially important roles during the development of landrace soybean lines. However, the elite MSU group showed favorable haplotypes at *qtlS.10* in only a small fraction of lines (0.141), confounding any conclusions regarding its role in soybean improvement. In general, far more variation was seen between *G.soja* and *G.max* than was seen between *G.max* subgroups when considering the *qtlP.20* and *qtlS.10* haplotypes, though this was not the case for the *qtlS.18* haplotypes.

## Conclusions

In this report, a preliminary haplotype-coupled QTL mapping analysis using a 210 RIL soybean population was conducted across 5 years for the interrogation of potential genomic regions associated with protein content and protein content stability. One QTL on chromosome 20 was found to explain approximately 20% variance in seed protein content, while stability analysis returned one QTL on chromosome 18 explaining approximately 7.6% PVE, and one QTL on chromosome 10 explaining approximately 8.6% PVE. Haplotype analysis using peak markers indicated the chromosome 20, 18, and 10 QTLs to have significant effects on observed phenotypes. The observed effects of the chromosome 18 and 10 markers on protein stability are noteworthy in that the response observed was independent of detectable shifts in protein content per se. Stacking desirable alleles of these QTL showed favorable genotypes to exhibit significantly improved protein content levels alongside improved stability, suggesting practical application for soybean performance enhancement. Prior to reliable application within established breeding pipelines, however, further interrogation within additional populations and environments will be necessary to examine the replicability of the results presented here.

## Supplementary Information

Below is the link to the electronic supplementary material.

Supplementary file1 (XLSX 265 kb)

## Data Availability

The phenotype and genotype data generated in this study are publicly available at https://github.com/AA-Mitchell/BARC6StabilityData. Additional information is available upon reasonable request.
